# Supplementary material for: Personal Care Product Use in Pregnancy and the Postpartum Period: Implications for Exposure Assessment
Source: Int J Environ Res Public Health. 2016 Jan 6;13(1):105. doi: 10.3390/ijerph13010105 (PMC4730496; doi:10.3390/ijerph13010105)
Supplement: Supplementary File 1 [file ijerph-13-00105-s001.pdf]

Supplementary Materials: Personal Care Product Use in Pregnancy and the Postpartum Period: Implications for Exposure Assessment

Carly Lang [1], Mandy Fisher [2,*], Angelica Neisa [2], Leona MacKinnon [2], Sandra Kuchta [2], Susan MacPherson [2], Adam Probert [2] and Tye E. Arbuckle [2]

Table S1. Unique PCP co-use combinations for participants during the weekday 48 h time period (T1A). 62 of the 63 participants reported PCP co-use (use of more than 1 PCP in a 48 h time period). There were 56 unique co-use combinations for the 62 participants.

PCP Combination	No. Users	% Users
anti, oral, condit, shampoo, bodysoap, soap, eyemakeup, makeup, face, lotion, hair, skinclea, lip	3	4.8
anti, oral, condit, shampoo, bodysoap, face, lotion, skinclea	2	3.2
anti, oral, condit, shampoo, bodysoap, eyemakeup, makeup, face, lotion, hair, skinclea	2	3.2
anti, oral, condit, shampoo, bodysoap, soap, face, skinclea	2	3.2
anti, oral, condit, shampoo, bodysoap, soap, eyemakeup, makeup, face, lotion, skinclea, lip	2	3.2
bodysoap, soap	1	1.6
oral, makeup, eyemakeup, skinclea	1	1.6
oral, bodysoap	1	1.6
oral, shampoo, soap, skinclea	1	1.6
oral, shampoo, bodysoap	1	1.6
oral, shampoo, bodysoap, eyemakeup, face, skinclea, lip	1	1.6
oral, shampoo, bodysoap, soap, eyemakeup, makeup, face, lotion, skinclea	1	1.6
oral, condit, shampoo	1	1.6
oral, condit, shampoo, bodysoap, eyemakeup, makeup, face, lotion, hair, skiclea, lip	1	1.6
oral, condit, shampoo, bodysoap, soap, lip	1	1.6
oral, condit, shampoo, bodysoap, soap, lotion, hair, skinclea	1	1.6
oral, condit, shampoo, bodysoap, soap, eyemakeup, face, lotion, skinclea	1	1.6
oral, condit, shampoo, bodysoap, soap, eyemakeup, makeup, face, hair	1	1.6
oral, condit, shampoo, bodysoap, soap, eyemakeup, makeup, face, lotion, skinclea	1	1.6
anti, condit, shampoo, bodysoap, soap, eyemakeup, makeup, face, lotion, hair	1	1.6
anti, oral, eyemakeup, makeup, lotion	1	1.6
anti, oral, soap, face, lotion, hair	1	1.6
anti, oral, soap, makeup, face, lotion, skinclea	1	1.6
anti, oral, bodysoap, soap, eyemakeup, makeup, face, lotion, hair, lip	1	1.6
anti, oral, shampoo, bodysoap, face, skinclea	1	1.6
anti, oral, shampoo, bodysoap, soap	1	1.6
anti, oral, shampoo, bodysoap, soap, skinclea	1	1.6
anti, oral, shampoo, bodysoap, soap, hair	1	1.6
anti, oral, shampoo, bodysoap, soap, lotion, skinclea	1	1.6
anti, oral, shampoo, bodysoap, soap, face	1	1.6
anti, oral, condit, shampoo, bodysoap, face, lotion, hair	1	1.6

Table S1. *Cont.*

PCP Combination	No. Users	% Users
anti, oral, condit, shampoo, bodysoap, face, lotion, hair, skinclea, lip	1	1.6
anti, oral, condit, shampoo, bodysoap, makeup, face, lotion	1	1.6
anti, oral, condit, shampoo, bodysoap, eyemakeup, face, lotion	1	1.6
anti, oral, condit, shampoo, bodysoap, eyemakeup, face, lotion, skinclea, lip	1	1.6
anti, oral, condit, shampoo, bodysoap, eyemakeup, makeup, face, lotion, hair, skinclea, lip	1	1.6
anti, oral, condit, shampoo, bodysoap, soap, skinclea	1	1.6
anti, oral, condit, shampoo, bodysoap, soap, lotion, skinclea	1	1.6
anti, oral, condit, shampoo, bodysoap, soap, face, lip	1	1.6
anti, oral, condit, shampoo, bodysoap, soap, face, hair	1	1.6
anti, oral, condit, shampoo, bodysoap, soap, face, lotion	1	1.6
anti, oral, condit, shampoo, bodysoap, soap, face, lotion, skinclea	1	1.6
anti, oral, condit, shampoo, bodysoap, soap, face, lotion, hair, skinclea	1	1.6
anti, oral, condit, shampoo, bodysoap, soap, makeup, face, skinclea	1	1.6
anti, oral, condit, shampoo, bodysoap, soap, makeup, face, lotion, hair	1	1.6
anti, oral, condit, shampoo, bodysoap, soap, makeup, face, lotion, skinclea, lip	1	1.6
anti, oral, condit, shampoo, bodysoap, soap, eyemakeup, face, lotion, hair, skinclea	1	1.6
anti, oral, condit, shampoo, bodysoap, soap, eyemakeup, makeup, hair, skinclea	1	1.6
anti, oral, condit, shampoo, bodysoap, soap, eyemakeup, makeup, lotion, hair	1	1.6
anti, oral, condit, shampoo, bodysoap, soap, eyemakeup, makeup, lotion, hair, skinclea, lip	1	1.6
anti, oral, condit, shampoo, bodysoap, soap, eyemakeup, makeup, face, lotion, lip	1	1.6
anti, oral, condit, shampoo, bodysoap, soap, eyemakeup, makeup, face, lotion, hair, lip	1	1.6
anti, oral, condit, shampoo, bodysoap, soap, eyemakeup, makeup, face, lotion, hair, skinclea	1	1.6

Table S2. Unique PCP co-use combinations for participants during the weekend 48 h time period (T1B). 66 of the 67 participants reported PCP co-use (use of more than 1 PCP in a 48 h period). There were 62 unique co-use combinations for the 66 participants.

PCP Combination	No. Users	% Users
anti, oral, condit, shampoo, bodysoap, soap, eyemakeup, makeup, face, lotion, hair, skinclea, lip	3	4.5
anti, oral, condit, shampoo, bodysoap, eyemakeup, makeup, face, lotion, hair, skinclea	2	3.0
oral, shampoo, bodysoap, soap	2	3.0
shampoo, bodysoap, lotion	1	1.5
condit, shampoo, bodysoap, eyemakeup, makeup, lotion, skinclea, lip	1	1.5
oral, face, lip	1	1.5
oral, eyemakeup, makeup, face, lotion, lip	1	1.5
oral, soap, face, lotion	1	1.5
oral, bodysoap, face, lotion, skinclea	1	1.5
oral, bodysoap, soap, eyemakeup, makeup, lotion, lip	1	1.5
oral, shampoo, eyemakeup, makeup, lotion, lip	1	1.5
oral, shampoo, soap, eyemakeup, makeup, face, lotion, hair, skinclea	1	1.5
oral, shampoo, bodysoap, face, lotion, hair, lip	1	1.5
oral, shampoo, bodysoap, soap, face, lotion, skinclea	1	1.5
oral, shampoo, bodysoap, soap, face, lotion, hair, skinclea	1	1.5
oral, condit, shampoo, eyemakeup, makeup, hair, skinclea	1	1.5
oral, condit, shampoo, eyemakeup, makeup, lotion, hair, skinclea	1	1.5
oral, condit, shampoo, soap, face, lotion, skinclea, lip	1	1.5
oral, condit, shampoo, soap, eyemakeup, lotion, lip	1	1.5
oral, condit, shampoo, bodysoap, makeup, face	1	1.5
oral, condit, shampoo, bodysoap, eyemakeup, lotion, lip	1	1.5
oral, condit, shampoo, bodysoap, eyemakeup, makeup, face, skinclea, lip	1	1.5
oral, condit, shampoo, bodysoap, eyemakeup, makeup, face, lotion, hair, skinclea	1	1.5
oral, condit, shampoo, bodysoap, eyemakeup, makeup, face, lotion, hair, skinclea, lip	1	1.5
oral, condit, shampoo, bodysoap, soap, lotion, skinclea	1	1.5
oral, condit, shampoo, bodysoap, soap, eyemakeup, makeup, skinclea	1	1.5
anti, shampoo, bodysoap, soap, eyemakeup, face, lotion, hair, skinclea	1	1.5
anti, oral, soap, lotion, skinclea	1	1.5
anti, oral, soap, lotion, hair, skinclea	1	1.5
anti, oral, soap, face, lotion	1	1.5
anti, oral, bodysoap	1	1.5
anti, oral, bodysoap, soap	1	1.5
anti, oral, shampoo, bodysoap, soap, lotion	1	1.5
anti, oral, shampoo, bodysoap, soap, lotion, skinclea	1	1.5
anti, oral, condit, shampoo, lotion, skinclea	1	1.5
anti, oral, condit, shampoo, bodysoap, skinclea	1	1.5
anti, oral, condit, shampoo, bodysoap, hair, skinclea	1	1.5
anti, oral, condit, shampoo, bodysoap, face, lotion	1	1.5
anti, oral, condit, shampoo, bodysoap, face, lotion, skinclea	1	1.5
anti, oral, condit, shampoo, bodysoap, face, lotion, hair, lip	1	1.5
anti, oral, condit, shampoo, bodysoap, face, lotion, hair, skinclea	1	1.5

Table S2. *Cont.*

PCP Combination	No. Users	% Users
anti, oral, condit, shampoo, bodysoap, makeup, lip	1	1.5
anti, oral, condit, shampoo, bodysoap, eyemakeup, face, lotion, skinclea	1	1.5
anti, oral, condit, shampoo, bodysoap, eyemakeup, makeup, lotion, skinclea, lip	1	1.5
anti, oral, condit, shampoo, bodysoap, eyemakeup, makeup, face, hair, lip	1	1.5
anti, oral, condit, shampoo, bodysoap, eyemakeup, makeup, face, lotion	1	1.5
anti, oral, condit, shampoo, bodysoap, soap, hair	1	1.5
anti, oral, condit, shampoo, bodysoap, soap, lotion	1	1.5
anti, oral, condit, shampoo, bodysoap, soap, face	1	1.5
anti, oral, condit, shampoo, bodysoap, soap, face, lotion	1	1.5
anti, oral, condit, shampoo, bodysoap, soap, face, lotion, lip	1	1.5
anti, oral, condit, shampoo, bodysoap, soap, face, lotion, skinclea	1	1.5
anti, oral, condit, shampoo, bodysoap, soap, makeup, lotion, lip	1	1.5
anti, oral, condit, shampoo, bodysoap, soap, makeup, lotion, skinclea	1	1.5
anti, oral, condit, shampoo, bodysoap, soap, makeup, face, lotion, skinclea, lip	1	1.5
anti, oral, condit, shampoo, bodysoap, soap, makeup, face, lotion, hair	1	1.5
anti, oral, condit, shampoo, bodysoap, soap, makeup, face, lotion, hair, skinclea, lip	1	1.5
anti, oral, condit, shampoo, bodysoap, soap, eyemakeup, face, lotion, skinclea	1	1.5
anti, oral, condit, shampoo, bodysoap, soap, eyemekaup, makeup, lotion, skinclea, lip	1	1.5
anti, oral, condit, shampoo, bodysoap, soap, eyemakeup, makeup, face, skinclea	1	1.5
anti, oral, condit, shampoo, bodysoap, soap, eyemakeup, makeup, face, lotion, skinclea	1	1.5
anti, oral, condit, shampoo, bodysoap, soap, eyemakeup, makeup, face, lotion, hair, skinclea	1	1.5

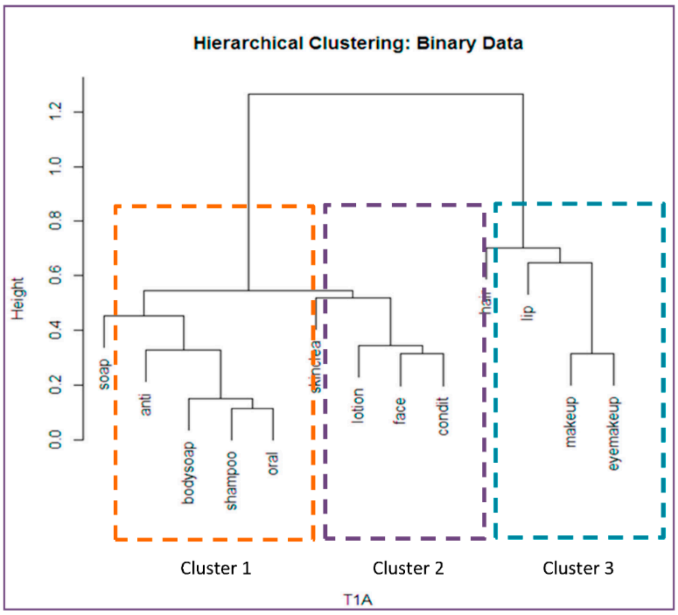

Figure S1. Dendrogram representing hierarchical clustering from study time period T1A weekday (48 h, weekdays, n = 63).

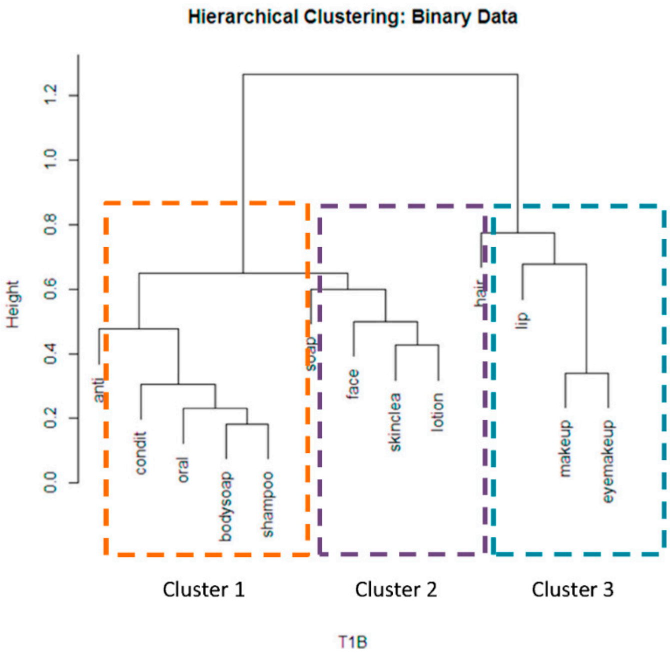

Figure S2. Dendrogram representing hierarchial clustering from study time period T1B weekend (48 h, weekend, $n = 67$).

Abreviations/Definitions

PCP: personal care product
T1A: first trimester study period, weekday
T1B: first trimester study period, weekend
makeup: general makeup and cosmetics
lip: lip products
eyemakeup: eye makeup and cosmetics
hair: hairstyling products
condit: conditioner
bodysoap: body soaps
skinclea: facial soaps, cleansers and washes
oral: toothpaste and mouthwash
soap: hand soaps, sanitizers and soap not otherwise specified
anti: deodorant and antiperspirants
lotion: body lotions, creams and oils
face: face lotions and creams

© 2016 by the authors; licensee MDPI, Basel, Switzerland. This article is an open access article distributed under the terms and conditions of the Creative Commons by Attribution (CC-BY) license (http://creativecommons.org/licenses/by/4.0/).
